# A Patient Portal With Electronic Messaging: Controlled Before-and-After Study

**DOI:** 10.2196/jmir.4487

**Published:** 2015-11-09

**Authors:** Iiris Riippa, Miika Linna, Ilona Rönkkö

**Affiliations:** ^1^ Department of Industrial Engineering and Management Aalto University Espoo Finland; ^2^ National Institute for Health and Welfare Helsinki Finland; ^3^ The City of Hämeenlinna Hämeenlinna Finland

**Keywords:** chronic illness, patient activation, self-management, cost-effectiveness, patient portal

## Abstract

**Background:**

Patients’ access to their medical records, along with electronic messaging, offers an efficient means of information transition between patients and their caregivers. Easier access to information and interaction with health care professionals may reduce use of other services while increasing patients’ activation in the management of their own health. Patient portals may therefore have a favorable impact on the cost-effectiveness of care.

**Objective:**

The aim was to assess the benefits and risks of providing electronic messaging services to patients with chronic conditions. Using cost-effectiveness analysis, the outcomes and costs of providing access to an electronic patient portal were evaluated in a real-life treatment process in primary care.

**Methods:**

A total of 876 chronically ill patients from public primary care were allocated to either an intervention group receiving immediate access to a patient portal that included their medical records, care plan, and secure messaging with a care team, or to a control group receiving standard care. Incremental direct heath care costs, health status based on the Short-Form Health Survey, version 2 (SF-36v2), and patient activation based on the short form of the Patient Activation Measure (PAM13) were compared to standard care in a 6-month follow-up. Incremental cost-effectiveness ratios were calculated using a sample of 80 patients in the intervention group and 57 patients in the control group; thus, a total of 137 patients were included in the final analysis. Propensity-score matching was used to assess the sensitivity of the results to the possible attrition bias.

**Results:**

Patient activation improved more in the intervention group but the effect was not statistically significant. The effect on cost of care was ambiguous; costs decreased by an average of €91 in the unadjusted model, but increased by €48 in the adjusted model. Due to the controversial results on cost, the unadjusted analysis showed an 89% probability of cost-effectiveness with no willingness to pay for increased patient activation, whereas in the adjusted sample, the probability of the portal being more cost-effective than care as usual exceeded 50% probability at a willingness to pay €700 per clinically significant increase in patient activation score. There was no marked short-term impact on health status based on the SF-36v2 measure.

**Conclusions:**

Offering the possibility to substitute health care visits with less costly contacts using self-management tools did not seem to compromise the health status or treatment of chronic care patients. Patient activation increased, and this could be achieved with moderate costs in a short-term experiment. In the long term, increased activation is proposed to lead to better health outcomes and eventually cut down resource use. Future studies should assess the long-term effects of patient portals on patients’ health status and cost of care.

## Introduction

Health maintenance and the restoration of functioning among the chronically ill requires repetitive interaction with the care provider and patient engagement in the management of their own condition [1]. To meet the needs of the growing population with chronic diseases, health care providers have begun efforts to engage chronically ill patients in monitoring and managing their own health. Supporting patients’ self-management may impact patients’ use of traditionally provided health services, but can also have an impact on health outcomes by increasing patients’ activation in the management of their own health [2]. Activated patients are knowledgeable, skilled, and confident in the management of their condition, and are shown to engage in preventive behavior by following care recommendations and pursuing healthy lifestyles [3-5].

An electronic patient portal is one of the self-management tools suggested for increasing patient activation [6,7] by enabling efficient information sharing between a patient and the health care provider, and by improving patient access to communication with a health care professional [8-10]. Patient portals typically provide the patient with access to their own medical health records documented and managed by a health care institution [8,11]. Other common patient portal functionalities are secured electronic messaging with a health care professional, medication refills, and access to medical information [11]. In addition to the potential positive effect on patient activation, a patient portal may also relieve the need for health services offered through traditional channels, such as phone calls and face-to-face office visits [12].

Along with these positive expectations for the effects of a patient portal, there have also been doubts whether physician or nurse visits can be substituted with self-management and electronic messaging without adverse effects on health outcomes. Some concern has been expressed about the loss of interpersonal relationships between the patient and the caregiver and on the possible worry that seeing one’s medical information might cause to the patient [13].

To decide on the adoption and implementation of new self-management tools, practitioners and policy makers need information on how effective—in terms of their impact on patient activation and health outcomes—the tools are relative to their impact on the cost of care provision. Previous studies on electronic patient portals have assessed either their effect on use of other health services [12,14] or their impact on care outcomes [7,15-17]. Limited attention has been paid to the simultaneous assessment of the additional costs and care outcomes of an electronic patient portal compared to standard care, namely the cost-effectiveness of an electronic patient portal. This paper reports the cost-effectiveness evaluation of an electronic patient portal conducted along with a controlled before-and-after study. To account for possible changes in patient activation and in patient-assessed state of health, we report cost-effectiveness comparisons for both of these care outcomes.

## Methods

### Study Setting, Participants, and the Intervention

**Figure 1:**
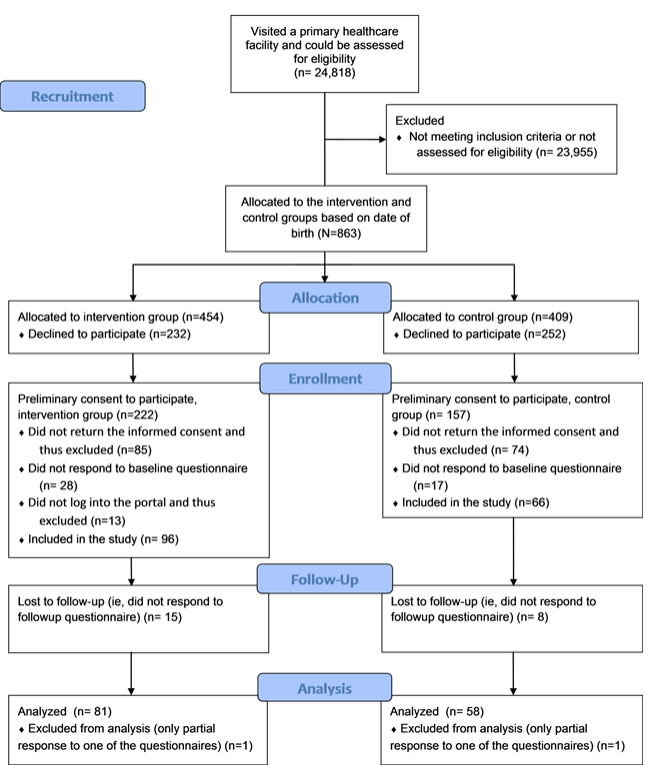
Patient flow.

The setting was a controlled before-and-after study conducted in Finnish public primary care in 2012 [18]. Patients were recruited to the study by nurses and doctors during their visit to primary care. To be included in the study, patients had to meet the following eligibility criteria: (1) age of at least 18 years, (2) at least two treatable health conditions assessed by a health professional, (3) bank identifiers (ie, electronic credentials for online authentication provided by their bank) and access to the Internet, and (4) be willing and able, both according to themselves and to a health care professional, to engage in using the portal. The eligible patients were allocated either to the intervention group or the control group on the basis of their date of birth. Patients born on odd dates were assigned to the intervention group, and patients born on even dates were assigned to the control group. The ethical board of the local authority (Pirkanmaa Hospital District) approved the study protocol, and informed consent was obtained from all patients included in the study (see Figure 1).

The intervention group received immediate access to the patient portal, and participants in the control group were to receive delayed portal access after 6 months. Each study participant formed a personally tailored care plan together with a health care professional. Whereas the intervention group was given online access to their care plan through the portal, patients in the control group received a printed copy of their plan. In addition to the electronic care plan, the patient portal contained access to (1) the patient’s own patient records, provided and maintained by the health care provider with diagnoses of chronic illnesses and permanent medication prescriptions, (2) laboratory results with statements from a health care professional, (3) vaccination history, and (4) electronic messaging with a health care professional. The names of diagnoses, medicines, and laboratory results were linked to relevant additional information in the online medical information service, Health Library [19], administered by The Finnish Medical Society, Duodecim. The users could visit the portal through the care provider’s webpages. For secure identification, the patient used his or her bank identifiers to sign in. The patient portal studied was relatively simple as it contained no features for patient-produced information of their health management (eg, see Nagykaldi et al [6]) or interactive condition-specific health education (eg, see Solomon et al [7]).

### Materials

#### Outcome Data

The first measure of effectiveness in this study was patient activation, assessed using the short form of the Patient Activation Measure (PAM13, see Multimedia Appendix 1). The PAM13 was created by Hibbard and colleagues [20] and measures patients' knowledge of their diseases, skills to self-manage their diseases, and self-confidence in their abilities to manage their diseases. The validity assessment of the Finnish translation for the PAM13 instrument has been reported elsewhere [18]. The instrument consists of 13 statements, which are answered by the respondents with degrees of agreement or disagreement. The raw PAM13 scores (range 13-52) were linearly converted to activation scores ranging from zero (lowest activation) to 100 (highest activation) following established Patient Activation Measure (PAM) methodology [21]. Increases in the patient activation score have been shown to be followed by improved health behaviors [4,22]; thus, the measure can be used as an intermediate outcome measure for self-management interventions [3].

The second measure of effectiveness was related to health status and quality of life. Here, patients self-assessed their physical and mental health based on the second version of the Short-Form Health Survey (SF-36v2). The Short-Form Health Survey (SF-36) was created by John E Ware and colleagues [23-25] to evaluate patient perception of their physical and mental well-being. In previous registered clinical trials, the SF-36 is the most broadly used instrument for evaluating patient-reported health outcomes in clinical trials [26]. Responses to the SF-36v2 were collected along with the PAM13 questionnaire from both the intervention and control groups at baseline and after 6 months from enrollment in the study. Age, gender, the number of chronic diseases, and the prevalence of the most common chronic conditions in the sample (ie, diabetes, hypercholesterolemia, and hypertension) were retrieved from the electronic patient records.

#### Costs and Resource Use Data

The cost of primary health care contacts during the 6 months before the intervention and in the 6 months following were calculated for each individual, as were the costs of providing access to the portal. The use of primary health care resources was collected directly from the patient administration system (PAS), which contained patient-level data abstracts from the electronic patient records. The PAS data included contact types—such as visits, phone calls, or electronic messaging—the patient’s age, the diagnoses (International Classification of Diseases, 10th revision [ICD-10]), the reason for the encounter (International Classification for Primary Care, version 2 [ICPC-2]), and the employee category of the health care professional in the contact.

Extracting the patient-level data from the patient administration systems—with diagnosis and activity information—made it possible to group each individual encounter type using the Ambulatory and Primary Care Related Patient Groups (APR) grouper software, a grouping system equivalent to diagnosis-related groups (DRG) used in hospital care [27]. The batch grouper software assigned each individual patient encounter to one of the 144 APR groups. After grouping, each of the 144 APR groups in the sample was assigned a cost weight, indicating the relative consumption of resources. APR cost weights were based on the same standard time measurements which are also  used in the national unit price lists for health and social services [28] published by the National Institute for Health and Welfare (THL). The resource consumption of messages sent via the patient portal was also included in the costs. A secure message to the patient using the patient portal was considered to require the same amount of a health care professional’s time as sending an email or a letter. Thus, the APR grouper used identical cost weights for these types of contacts.

The cost of providing access to the patient portal for this study was €6 per year per patient. This estimate was based on a 5-year depreciation plan of the portal deployment cost, on the expected average number of users during this period, and on the yearly maintenance cost (see Table 1). The number of patient portal users in 2014 after 2 years from portal adoption was 3527, and the number was expected to grow by 1000 users per year in 2015 and 2016.

**Table 1:** Cost of the patient portal.

Components of cost	Year					5-year average
	2012	2013	2014	2015	2016	
Depreciation of the deployment cost (€)	3095	3095	3095	3095	3095	3095
Maintenance cost (€)	7068	11,120	11,902	12,780	13,380	11,250
Total cost (€)	10,163	14,215	14,997	15,875	16,475	14,345
Number of users, n	760	2315	3527	4527	5527	3331
Cost per user (€)	13.4	6.1	4.3	3.5	3.0	6.0

### Statistical Analysis

To examine the similarity of the intervention and control groups at baseline, we tested for differences in age, gender, number of chronic diseases, prevalence of the most common chronic conditions in the sample (ie, diabetes, hypercholesterolemia, and hypertension), doctor and nurse visits, cost of care, physical and mental health, and patient activation. Independent-sample *t* tests for continuous variables and chi-square tests for categorical variables were used.

Due to the possible selection bias resulting from the nonblinding of the participants [18], we used propensity-score matching [29] to adjust for the baseline differences between the intervention and control groups. Here, the propensity score is the predicted value from logistic regression, with portal access as the dependent variable. Brookhart and colleagues [30] advise to include the variables that may affect the outcomes of interest in the logistic regression. Therefore, in addition to age and gender, baseline measurements for all outcome variables followed; the cost of care, patient activation, and patient-reported health indicators were included in the logistic regression. After calculating the propensity scores, nearest-available matching [29] was used to pair each participant in the control group with a participant in the intervention group based on the propensity score similarities.

To assess the achieved balance between the matched groups, we tested for the standardized differences for each covariate at baseline. Standardized difference is the difference in means between the two groups in units of standard deviation [31]. A value of less than 20% is considered to indicate an adequate balance and therefore good comparability between the groups [29].

To assess cost-effectiveness of the intervention, we used nonparametric bootstrapping to simulate 1000 incremental cost-effectiveness ratios (ICERs) for both the matched and nonmatched samples and plotted them on a cost-effectiveness plane. This method is widely used in health economics evaluations (eg, see Bos et al [32] and van Spijker et al [33]) to study the health effects of an intervention in relation to the cost of care induced by the intervention [34]. Here, ICER is the ratio between the incremental cost and the incremental effectiveness, which can be changes in patient activation or in health status. Each bootstrapped ICER falls into one of the four quadrants of the cost-effectiveness plane where differences in average effectiveness are displayed on the x-axis and differences in average costs on the y-axis. The quadrants represent four possible situations in relation to the incremental cost and incremental effectiveness of the intervention in comparison with care as usual. The proportion of bootstrapped ICERs that fall into a quadrant indicates the likelihood of the outcome represented by the quadrant. In addition to the cost-effectiveness plane, we calculated the cost-effectiveness acceptability curves (CEACs) for patient portal cost-effectiveness [35]. The acceptability curve indicates the probability for cost-effectiveness of the intervention at different levels of willingness to pay for the additional health outcome [36].

## Results

### Descriptive Characteristics

There were no significant differences in the baseline characteristics between the intervention and control groups (see Table 2). A slightly greater proportion of the patients in the intervention group were women (45/80, 56%) compared to the control group (26/57, 46%). The mean cost of care during the year before the intervention was somewhat higher for the intervention group (€935) in comparison to the control group (€756). Patient-reported physical and mental health and patient activation at baseline were similar in both groups.

**Table 2:** Descriptive characteristics of study participants.

Characteristic		Portal access (n=80)	Control (n=57)	*t*_135_	χ^2^_1_	*P*
Age (years), mean (SD)		61 (9)	63 (10)	-0.8		.40
Female, n (%)		45 (56)	26 (46)		1.5	.22
Number of chronic diagnoses^a^, mean (SD)		1.3 (1.3)	1.4 (1.4)	-0.6		.53
**Diagnosis, n (%)**						
	Type 1 or 2 diabetes^a,b^	32 (40)	22 (39)		0	.87
	Hypertension^a,c^	22 (28)	21 (37)		1.3	.25
	Hypercholesterolemia^a,d^	37 (46)	24 (42)		0.2	.63
Doctor visits^e^, mean (SD)		3.8 (3.3)	3.0 (3.1)	1.4		.18
Nurse visits^e^, mean (SD)		3.5 (2.6)	4.1 (2.5)	-1.3		.18
Cost of care^e^ (€), mean (SD)		935 (767)	756 (528)	1.5		.13
Patient activation, mean (SD)		63.7 (15.4)	63.4 (14.5)	0.1		.89
SF-36v2^f^ Physical Health subscale, mean (SD)		65.9 (19.3)	63.8 (20.6)	0.6		.55
SF-36v2 Mental Health subscale, mean (SD)		72.8 (21.1)	73.5 (19.6)	-0.2		.85

^a^From the time before the beginning of the intervention.

^b^International Classification of Diseases, 10th revision (ICD-10) codes E10-E14 or International Classification for Primary Care, version 2 (ICPC-2) codes T89-T90.

^c^ICD-10 codes I10-I15 or ICPC-2 codes K85-K87.

^d^ICD-10 code E78 or ICPC-2 code T93.

^e^During the year before the intervention.

^f^SF-36v2: Short-Form Health Survey, version 2.

### Propensity-Score Matching

The matching reduced the sample size to 114 participants, with 57 participants in each of the intervention and control groups. Before matching, the standardized difference was over 20% for two of the covariates, namely gender and cost of care. After matching, this statistic was below 15% for each covariate, suggesting that the matching was successful (see Table 3). Standardized difference calculations for variables were based on Austin [37].

**Table 3:** Covariables at baseline before and after matching.

Characteristic	Before matching			After matching		
	Portal access (n=80)	Control (n=57)	Standardized difference^a^	Portal access (n=57)	Control (n=57)	Standardized difference^a^
Female, n (%)	45 (56)	26 (46)	21.2	26 (46)	26 (46)	0
Age, mean (SD)	61 (9)	63 (10)	-14.4	63 (7)	63 (10)	4.9
Cost of care^b^ (€), mean (SD)	584 (516)	468 (377)	25.8	455 (327)	468 (377)	-2.8
Patient activation, mean (SD)	63.7 (15.4)	63.4 (14.5)	2.3	65.2 (15.7)	63.4 (14.5)	12.3
Physical health, mean (SD)	65.9 (19.3)	63.8 (20.6)	10.4	63.7 (20.1)	63.8 (20.1)	-0.4
Mental health, mean (SD)	72.8 (21.1)	73.5 (19.6)	-3.4	73.1 (21.1)	73.5 (19.6)	-1.7

^a^Standardized difference for continuous variables = 100(x_i_-x_c_)/(s_i_^2^+s_c_^2^)^1/2^, where x_i_and x_c_are sample means in the intervention and control groups, respectively, and s_i_^2^and s_c_^2^are sample variances in the intervention and control groups, respectively. Standardized difference for dichotomous variables = 100(P_i_-P_c_)/{[P_i_(1-P_i_)+P_c_(1- P_c_)]/2}^1/2^where P_i_and P_c_denote the prevalence or mean of the dichotomous variable in treated and untreated subjects, respectively [37].

^b^During 6 months before the intervention.

### Unadjusted and Adjusted Effects of Portal Access on Cost of Care, Patient Activation, and Patient-Reported Health

In both matched and unmatched samples, none of the incremental changes in effectiveness measures and cost were statistically significant. In the adjusted sample, the incremental change in costs due to the intervention was €45 (95% CI -94 to 183), whereas in the unadjusted sample the incremental change in costs changed sign, being -€94 (95% CI -253 to 65). Results on patient activation and patient-reported health were less sensitive to the matching. The mean change in the patients’ activation score was 2.8 points (95% CI -2.2 to 7.8) higher in the intervention group, compared to the control group in the adjusted sample, and 2.6 points (95% CI -1.8 to 7.1) higher in the intervention group, compared to the control group in the unadjusted sample. The difference of 4-5 points in patient activation is considered clinically meaningful in terms of patients' health behavior [38,39]. The proportion of patients with clinically meaningful change (≥5 points) in patient activation was 7.0% higher in the intervention group in the matched sample and 5.7% higher in the intervention group in the unmatched sample. Differences in patient-reported physical and mental health changes were minor and changed sign from the matched (Physical Health, mean 1.2, 95% CI -3.3 to 5.7; Mental Health, mean 0.8, 95% CI -3.6 to 5.2) to the unmatched sample (Physical Health, mean -0.4, 95% CI -4.7 to 3.9; Mental Health, mean -0.4, 95% CI -4.8 to 4.0). Previous studies on the SF-36 Physical and Mental Health subscales have suggested a change of 4-5 points to be clinically significant in these measures [40,41] (see Table 4).

**Table 4:** Changes in outcome measures and incremental change due to the intervention: matched and unmatched samples.

Outcome	Portal access, matched (n=57)	Portal access, unmatched (n=80)	Control (n=57)	Incremental change, matched sample (n=114)	Incremental change, unmatched sample (n=137)
Cost of care (€)^a^, mean difference (95% CI)	39 (-61 to 140)	-99 (-215 to 16)	-9 (-106 to 89)	48 (-91 to 186)	-91 (-250 to 68)
Patient activation, mean difference (95% CI)	1.2 (-2.3 to 4.8)	1.1 (-1.8 to 3.9)	-1.6 (-5.2 to 2.1)	2.8 (-2.2 to 7.8)	2.6 (-1.8 to 7.1)
Patient activation, proportion of responders^b^, n (%) or %	20 (35)	27 (34)	16 (28)	7.0	5.7
Physical Health score, mean difference (95% CI)	-0.6 (-4.2 to 3.1)	-2.2 (-5.4 to 0.9)	-1.8 (-4.5 to 0.9)	1.2 (-3.3 to 5.7)	-0.4 (-4.7 to 3.9)
Mental Health score, mean difference (95% CI)	2.1 (-1.0 to 5.1)	0.9 (-2.1 to 3.8)	1.3 (-1.9 to 4.5)	0.8 (-3.6 to 5.2)	-0.4 (-4.8 to 4.0)

^a^Difference in cost of care 6 months before and after the intervention.

^b^Improvement of ≥5 points (scale 0-100).

### The Cost-Effectiveness Analysis

**Figure 2:**
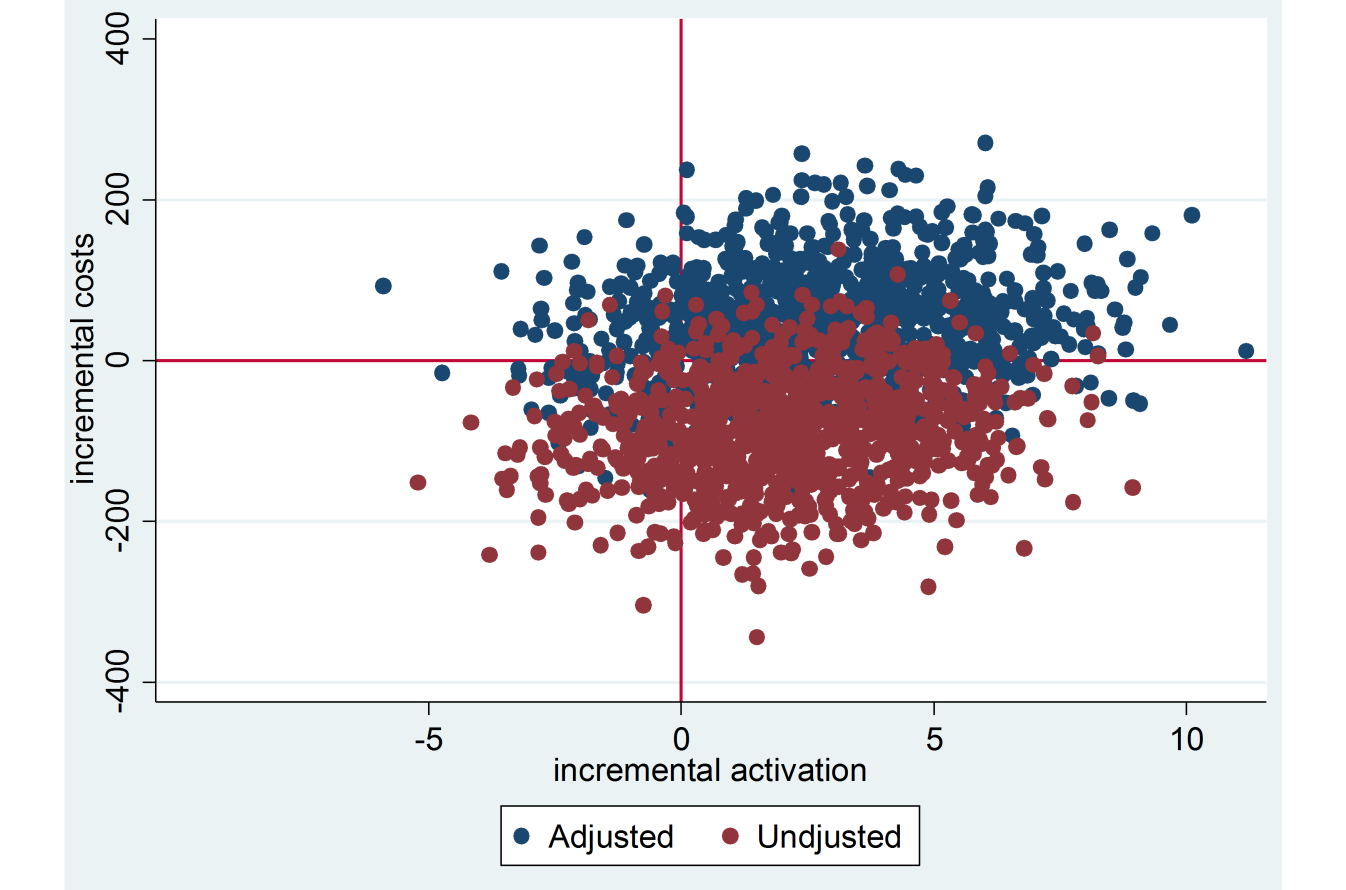
Distribution of bootstrapped incremental costs and activation with and without propensity-score matching adjustment.

The cost-effectiveness plane for patient activation after patient portal access shows greater uncertainty for the cost-effectiveness of the intervention in the matched sample compared to the unmatched sample. In the matched sample, 67.4% of the bootstrapped ICERs fall into the northeast quadrant, indicating increased activation at an incremental cost. In addition, 19.2% of the points fall into the southeast quadrant, 5.4% into the southwest quadrant, and 8.0% into the northwest quadrant. In the unadjusted sample, 71.9% of the simulated ICERs fall into the southeast quadrant, indicating that increased activation was generated with less cost by the intervention in comparison with care as usual (dominance). In addition, 9.4% of the ICERs fall into the northeast quadrant, suggesting increased activation at incremental cost, and 17.5% into the southwest quadrant, suggesting decreased activity at a lower cost. Only 1.2% of the data points fall into the northwest quadrant, suggesting a very small probability for decrease in patient activation at an incremental cost (see Figure 2).

**Figure 3:**
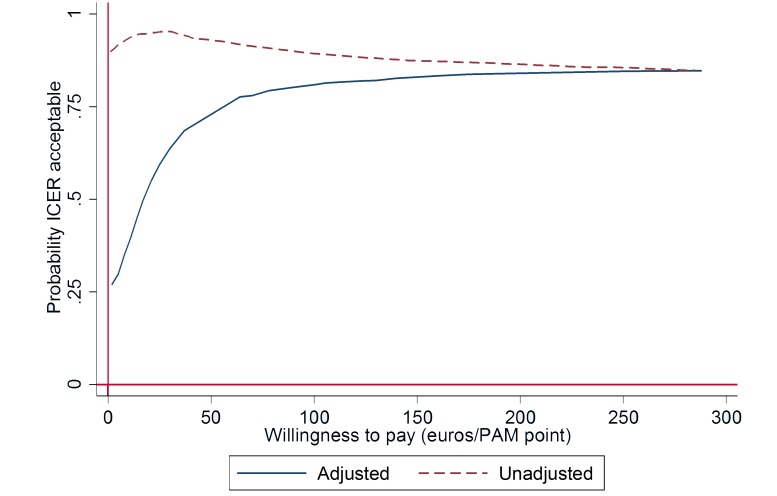
ICER acceptability curve based on willingness to pay for clinically significant change in patient activation gain.

The incremental cost-effectiveness acceptability curve (see Figure 3) for the matched sample shows that at a willingness to pay €18 per 1-point increase in patient activation, there is a 50% probability that the intervention is cost-effective. At a willingness to pay €40 per 1-point increase, the probability is 70%. In the unadjusted sample, at no willingness to pay for incremental patient activation points, the probability of intervention cost-effectiveness is 89%.

We also conducted a cost-effectiveness analysis for the proportion of clinically significant changes in patient activation (≥5-point increase). The results were parallel with the analysis for a 1-point increase in patient activation. In the adjusted sample, a majority (61.1%) of the bootstrapped ICERs fall into the northeast quadrant, indicating increased activation at an incremental cost. In the unadjusted sample, 57.7% of the simulated ICERs fall into the southeast quadrant, indicating that increased activation was generated for less cost by the intervention, in comparison with care as usual (dominance) (see Multimedia Appendix 2A). The incremental cost-effectiveness acceptability curve (see Multimedia Appendix 2B) for the adjusted sample shows that at a willingness to pay €700 per clinically significant change in patient activation, there is over 50% probability that the intervention is cost-effective. At a willingness to pay €2100 per clinically significant change in patient activation, the probability of cost-effectiveness rises to 70% in the adjusted sample. In the unadjusted sample, the probability of cost-effectiveness is 89% at a willingness to pay €0 per clinically significant change in patient activation. At a willingness to pay €2000 per clinically significant change in the PAM13, the acceptability was still as high as 82% in the unadjusted sample.

The cost-effectiveness planes for physical and mental health scores after patient portal access showed similar percentages of ICERs in the right-hand and left-hand quadrants, which confirms that there was no difference in these outcome measures between the intervention and care as usual (see Multimedia Appendices 2C and Multimedia Appendices 2D).

## Discussion

### Principal Findings

While patient activation increased more in the intervention group than in the control group, no significant effect of access to the patient portal on patient activation was detected in this study. This finding differs from previous findings on electronic patient portals [6,7]. Previous studies have also shown decreases in face-to-face visits due to patient portal use [6,12,42]. In this study, however, the effect of the portal access on cost of care was ambiguous, changing from more to less costly depending on the model used. Patient activation improved in both intervention and control groups. A plausible explanation for this may be the additional intervention delivered to both the intervention and control groups, namely the drafting of the care plan. Another explanation could be the patient activation survey itself, as it might encourage patients to rethink their role in the management of their condition.

Empirical investigations on electronic patient portals have predominantly assessed either their effect on the use of other health services [12,14] or their impact on care outcomes [7,15-17]. Only a few studies have assessed both the effects on care outcomes and service use [6] and, to our knowledge, this is the first study to assess the cost-effectiveness of a patient portal.

The results of the cost-effectiveness analysis, in which both effects on patient activation and cost of care were assessed simultaneously, show some support for the cost-effectiveness of a simple electronic patient portal that provides patients access to their own health records and secure messaging with the health care provider. Although no statistically significant improvement (>90% probability) in cost-effectiveness was detected in the sample adjusted for plausible attrition bias, the results indicated over 50% probability for cost-effectiveness of the intervention at a willingness to pay €18 per 1-point increase in the patient activation score. For a clinically significant improvement in patient activation, that is, a more than 5-point increase in patient activation score, the probability exceeded 50% probability at €700 per clinically significant change. To assess whether these investments in patient activation are acceptable from the service provider’s perspective, information on the consequences of the improvements in patient activation are needed.

In previous research, an increase in activation score has been shown to result in improved self-management behavior [4] and better health outcomes [3]. Further, an association between low patient activation and high cost of care [43], as well as between high patient activation and low cost of care after 2 years of activation assessment [44], have been found. Although the causal relationship between patient activation change and the change in cost of care has not been studied to date [44], increased patient activation may, indeed, decrease cost of care in the long term. These findings suggest that monetary investments in activating patients may well be acceptable in terms of the achieved patient activation and the proposed following health outcomes. Further longitudinal studies are needed to set acceptable thresholds for willingness to pay for improvements in patient activation.

### Strengths and Limitations

The main strengths of this study are the experimental setting with longitudinal design, the simultaneous assessment of cost and effectiveness of the intervention, and the use of scientifically validated measures for assessment of (1) patient-reported health outcomes (SF-36v2) and (2) patient activation (PAM13). Patient activation serves as “an intermediate outcome of care that is measurable and linked with improved [health] outcomes” [3].

Our study also has several limitations. As the service was to be offered in a timely manner to all of the customers of the target-study organization, time periods for the recruitment and follow-up were limited. Sample size remained modest, and this likely reduced the statistical significance of the effects. Further, the 6-month follow-up period might have been too short to capture the full benefits of the portal. According to the professionals working in the study organization, both professionals and patients spent part of the intervention time learning how to use the portal effectively, despite the fact that a small-scale pilot study with a restricted group of patients had been organized to test the portal before this investigation began.

In this study, the patients and the study recruiters (nurses and physicians) could not be blinded from the allocation of the participants to the intervention and control groups, and this has plausibly caused attrition bias in the study arms [18]. Whereas blinding the patients from receiving the intervention would solve the attrition bias problem, it may be challenging to execute in a self-management intervention study where patients are active participants in the intervention and when informed consent from the patient is required for ethical approval. In Web-based intervention studies, Samoocha and colleagues [45] suggest the use of “sham” websites to blind participants from not receiving the actual intervention. In this study, a “sham” portal could not be offered for practical reasons. To control for the plausible attrition bias [18], propensity-score matching, a widely used statistical method for reducing the effects of confounding in observational studies [37], was applied.

In this study, socioeconomic factors were not controlled for and this may compromise the generalization of the study findings in populations with highly varying socioeconomic status. Sarkar and colleagues [46] found that patients with lower levels of education may be less likely to use patient portals. In this study, each eligible participant was explicitly offered access to the portal and support for portal use was available, if needed. This may have mitigated some differences in patients’ ability and willingness to participate and use the portal. The usability of the portal or patient perceptions of the portal content were not assessed in this study. Further research should address the implementation of patient portals in health service organizations and assessments of patient and care team perceptions of the portal to better understand why some patient portals may be cost-effective and some not. Similarly, further research should investigate who benefits most from access to and use of patient portals. A previous study published on this topic found that the portal had the greatest effect on activation among patients starting at the highest level of patient activation [18]. It has also been reported previously [47] that previous care received by the patient, rather than state of health, age, gender, or patient activation, is an important factor predicting the attractiveness of electronic patient portal use. Among patients with similar disease burden, those who chose not to use the portal had received more services from primary care in the previous year than those who had used the portal [47].

Finally, when assessing cost of care, only the costs for the providing primary care organization could be assessed in this study. Access to an electronic patient portal may also have a comparative advantage in terms of opportunity cost to the patients’ time.

### Conclusions

In this controlled before-and-after study with 6-month follow-up, the effect of an electronic patient portal with secured messaging on an intermediary health outcome, patient activation, and cost of care were assessed to evaluate the cost-effectiveness of the intervention. The results suggest that a patient portal with secured messaging may be more cost-effective than care as usual among chronically ill patients. Considering the favorable effect of patient activation on patients’ final health outcomes and cost of care shown in previous research, increased patient activation was gained with moderate cost in this study. Further, no reverse effect of the intervention on patient-reported health was detected.

As efforts are made to increase patients’ active participation in the management of their own care, suitable and valid measures for assessing the short-term effectiveness of self-management interventions, such as the Patient Activation Measure, are needed. These measures for “intermediary health outcomes” should be validated by longitudinal studies with several years of follow-up that can grasp the causalities between both the intermediary and final health outcomes, and between the intermediary health outcomes and cost of care. This will aid in setting acceptable thresholds for willingness to pay for intermediary health outcomes.

## Appendix

Multimedia Appendix 1 The short form of the Patient Activation Measure (PAM13).

Multimedia Appendix 2 (A) Distribution of bootstrapped incremental costs and proportion of significant changes in activation with and without propensity-score matching adjustment. (B) ICER acceptability curve based on willingness to pay for a clinically significant change in patient activation gain. (C) Distribution of bootstrapped incremental costs and Mental Health subscale score with and without propensity-score matching adjustment. (D) Distribution of bootstrapped incremental costs and Physical Health subscale score with and without propensity-score matching adjustment.

## Abbreviations

APR: Ambulatory and Primary Care Related Patient Group
CEAC: cost-effectiveness acceptability curve
DRG: diagnosis-related group
ICD-10: International Classification of Diseases, 10th revision
ICER: incremental cost-effectiveness ratio
ICPC-2: International Classification of Primary Care, version 2
PAM: Patient Activation Measure
PAM13: the short form of the Patient Activation Measure
PAS: patient administration system
Pc: the prevalence or mean of the dichotomous variable in untreated subjects
Pi: the prevalence or mean of the dichotomous variable in treated subjects
sc2: sample variance in control group
SF-36: Short-Form Health Survey
SF-36v2: Short-Form Health Survey, version 2
si2: sample variance in intervention group
THL: National Institute for Health and Welfare
xc: sample mean in control group
xi: sample mean in intervention group
