# Supplementary figures and images for: A Patient Portal With Electronic Messaging: Controlled Before-and-After Study
Source: J Med Internet Res. 2015 Nov 9;17(11):e250. doi: 10.2196/jmir.4487 (PMC4642411; doi:10.2196/jmir.4487)

## Multimedia Appendix 2.

(A)

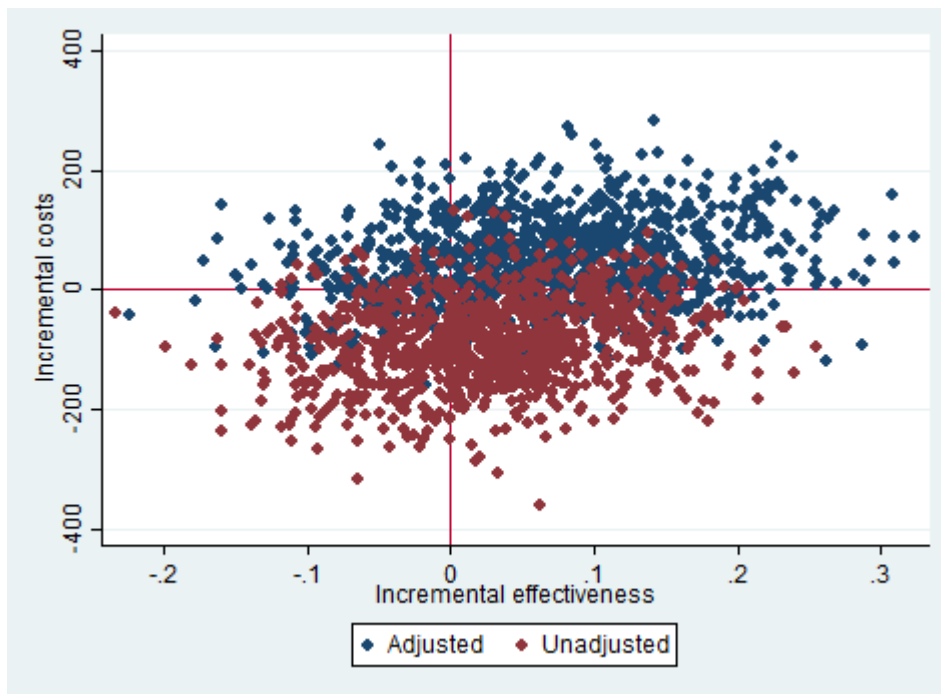

(B)

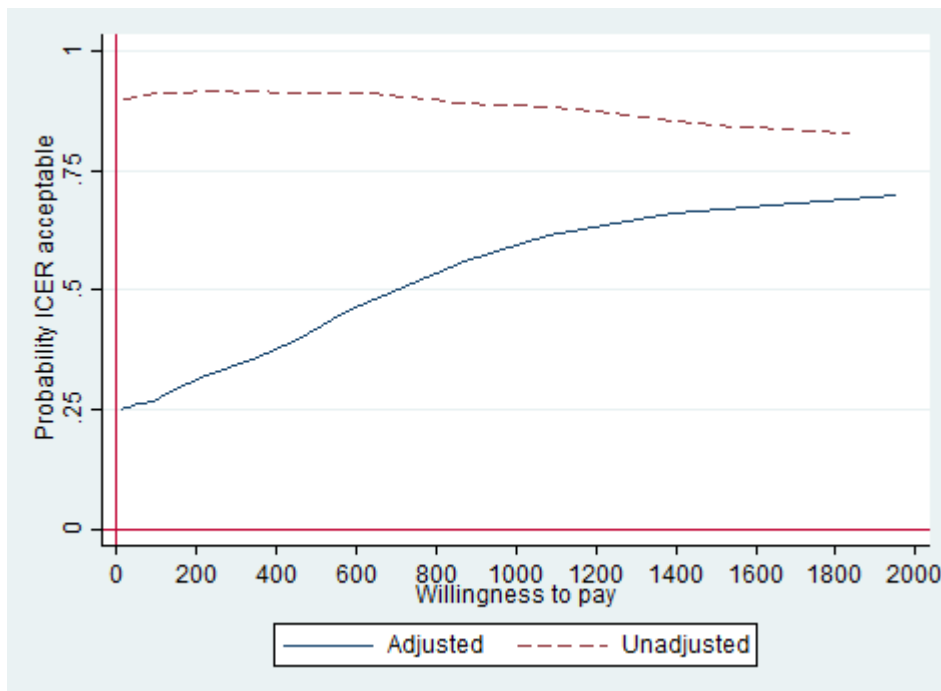

(C)

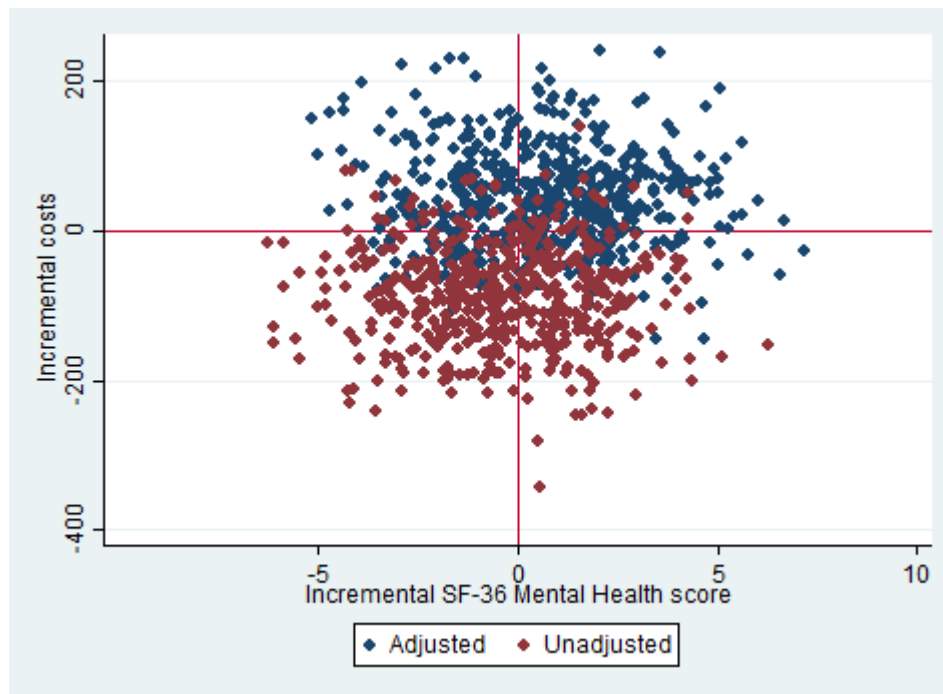

(D)

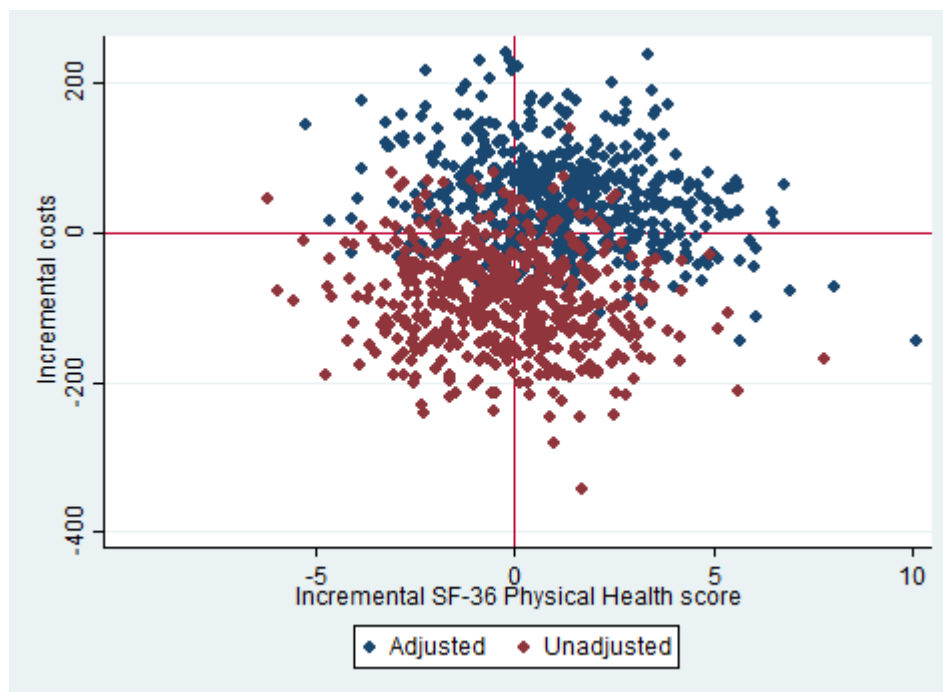

Supplement: Multimedia Appendix 2 [file jmir_v17i11e250_app2.pdf]
